# Acute abdomen due to anaphylactic intestinal edema associated with systematic mastocytosis: a case report

**DOI:** 10.1186/s12245-022-00441-5

**Published:** 2022-08-24

**Authors:** Tomoko Takagishi, Katsuhiko Miki, Shinsaku Imashuku, Katsushige Takagishi

**Affiliations:** 1 Department of Surgery, Ikoma City Hospital, Ikoma, Nara, 630-0213 Japan; 2 Department of Laboratory Medicine, Uji-Tokushukai Medical Center, Uji, Kyoto, 611-0041 Japan; 3 Department of Rheumatology, Nara City Hospital, Nara, 630-8305 Japan

**Keywords:** Acute abdomen, Systemic mastocytosis, Anaphylaxis, Intestinal edema

## Abstract

**Background:**

Among various anaphylactic conditions resulting in acute abdomen, mast cell activation disorders, although rare, are included in the differential diagnosis.

**Case presentation:**

This report describes a 63-year-old Caucasian man who was brought to the emergency room with sudden onset abdominal pain, vomiting, and diarrhea, with breathing difficulty, and with facial swelling after quarrelling with an acquaintance. Computed tomography showed edematous and swollen intestines, consistent with splenomegaly. Physical findings included maculopapular cutaneous mastocytosis. He also had a long history of repeated episodes of anaphylaxis requiring occasional epinephrine auto-injector administration; however, the precise cause of anaphylaxis was previously undetermined. Blood tests showed high serum concentrations of soluble IL-2R and tryptase, suggesting mast cell-related disease. Subsequent biopsies of his bone marrow and cutaneous rash confirmed the diagnosis of systemic mastocytosis (SM).

**Conclusion:**

SM was diagnosed in a patient with acute abdomen who visited the emergency room.

## Background

Acute abdomen in patients who visit the emergency room could be due to various types of gastrointestinal disorders [1]. A complete medical history and thorough physical examination are required in the differential diagnosis of acute abdomen. Anaphylaxis is one of the causes. In the differential diagnosis of anaphylactic diseases, patients showing persistent gastrointestinal symptoms together with skin and/or mucosa, respiratory, and systemic signs are classified as group 2 in the NIAID/FAAN criteria [2]. Thus, it is possible that unexplained acute abdomen may result from any types of anaphylactic conditions, either by mast cell-derived histamine or by bradykinin in cases of mast cell-related disorders [3] or of hereditary angioedema, a rare variant of bradykinin-mediated angioedema [4]. Mast cell activation disorders, involving clonal proliferation of abnormal mast cells, are well known to develop cutaneous disease like maculopapular cutaneous mastocytosis (MPCM) and/or systemic mastocytosis (SM) [5–7], in whom besides skin rashes, anaphylactic flushing and gastrointestinal symptoms often occur repeatedly.

As abdominal signs/symptoms in SM, patients may present with chronic or acute abdominal cramps, nausea/ vomiting, and diarrhea as vague or subtle manifestations. Causes of abdominal pain in SM are thought to be due to physical stimuli due to mast cell infiltration of the intestines, of the liver and spleen, and/or due to the release of chemical mediators such as histamine [8, 9]. Some patients may also present with symptoms of appendicitis or epiploic appendagitis [10, 11]. This report describes a 63-year-old man who presented to the emergency room with acute abdomen and was eventually diagnosed as SM.

## Case presentation

A 63-year-old Caucasian man, 175 cm in height and 75 kg in weight, was brought to the emergency room with sudden onset of abdominal pain, vomiting, and diarrhea, with breathing difficulty, and with facial swelling after quarrelling with an acquaintance. The medical history of this patient included repeated anaphylactic symptoms after eating pasta, drinking beer, taking loxoprofen, or at the time of feeling anxiety, having quarrels, etc. for more than 30 years, for which he occasionally received epinephrine auto-injector administration. He also had undergone detailed checkups for skin lesions and unexplained splenomegaly at other hospitals. A skin biopsy specimen obtained 3 years earlier showed the presence of nonspecific inflammatory cells but was nondiagnostic, whereas a bone marrow aspiration procedure resulted in dry tap with reticular fiber hyperplasia, suggesting primary myelofibrosis.

On admission to our hospital, his body temperature was 38.2 °C, his blood pressure was 106/88 mm/Hg, heart rate was 168 beats/min, and oxygen saturation was 80% on room air. Laboratory data showed a white blood cell count of 12,100/μL, consisting of 24.2% neutrophils, 72.1% lymphocytes, 1.9% monocytes, 1.7% eosinophils, and 0.1% basophils, with no abnormal cells. His hemoglobin concentration was 10.6 g/dL, his platelet count was 183 × 10^3^/μL, and his serum C-reactive protein concentration was 1.74 mg/dL. Evaluation of serum concentrations of hepatic enzymes revealed the following: lactate dehydrogenase, 96 U/L; aspartate aminotransferase, 31 U/L; alanine aminotransferase, 26 U/L; alkaline phosphatase, 174 U/L; and γ-glutamyl transpeptidase, 209 U/L. His renal function was normal. His serum concentrations of soluble interleukin-2 receptor (soluble IL-2R, 3695 U/mL; reference, 157–474 U/mL) and tryptase (444 µg/L; reference, 2.1–9.0 µg/L) were markedly higher than normal.

**Fig. 1:**
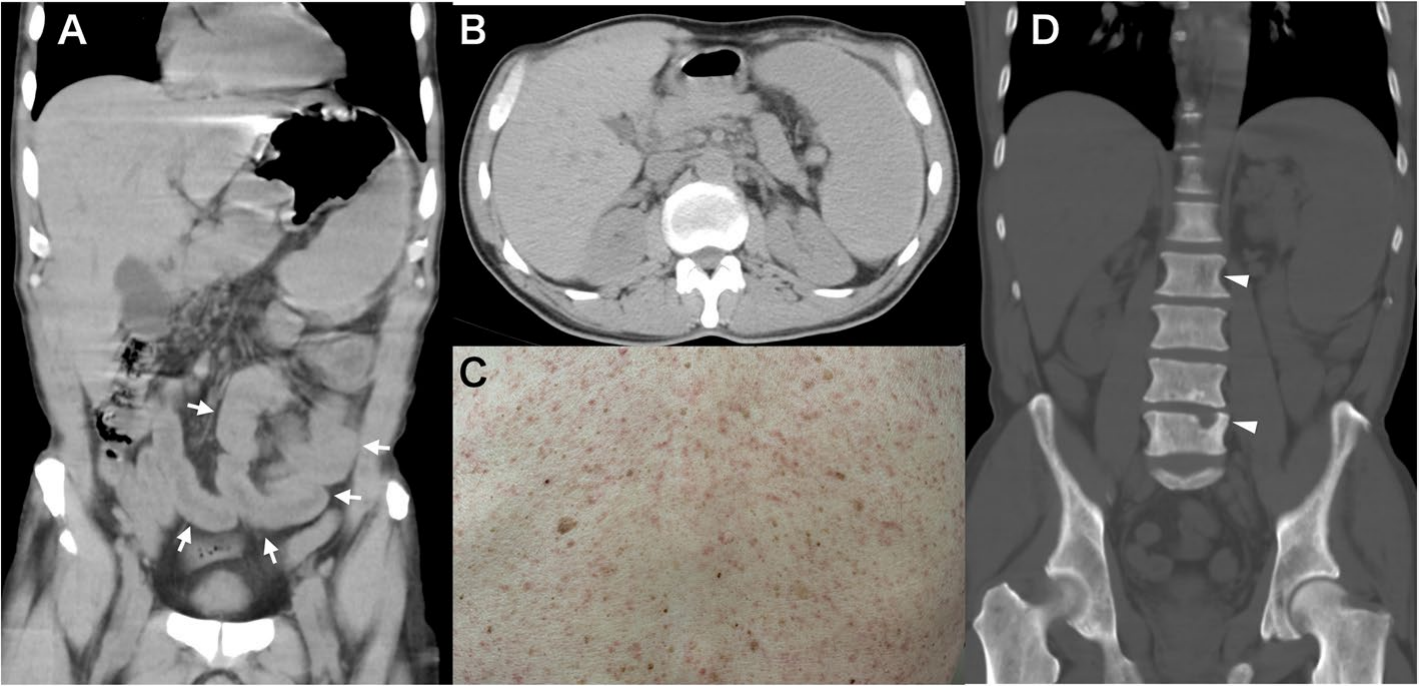
Abdominal CT of the patient showing **A** an edematous and swollen small intestine (arrows), **B** splenomegaly, and **C** maculopapular cutaneous mastocytosis on the back. **D** Bone density CT showing osteosclerosis of the pelvic bones, with partial punched out osteopenia of the spine (arrowheads)

Computed tomography (CT) of the abdomen at the emergency room showed a characteristically swollen and edematous small intestine (arrows) (Fig. 1A) and splenomegaly (Fig. 1B), but no evidence of mechanical obstruction. Physical examination showed diffuse MPCM on his trunk (chest, abdomen, and back), arms, and legs (Fig. 1C), which he had had for more than 30 years. Bone abnormality included osteopenia/osteoporotic lesions of the pelvic bones and in a few spinal bones (arrowheads) (Fig. 1D). Based on these results, he was suspected as having a possible anaphylactic combination of persistent gastrointestinal tract problems, symptoms of respiratory compromise, and involvement of the skin [2] associated with additional bone disease. He was treated with oxygen (7 L/min), intravenous saline containing Polaramine® (d-chlorpheniramine maleate; 5 mg/dose), Gaster® (famotidine; 20 mg/dose), and Solu-Medrol® (methylprednisolone sodium succinate;125 mg/dose). The abdominal cramps disappeared within 6 h. He became free from oxygen in about 3 h and was discharged from the hospital the next day.

**Fig. 2:**
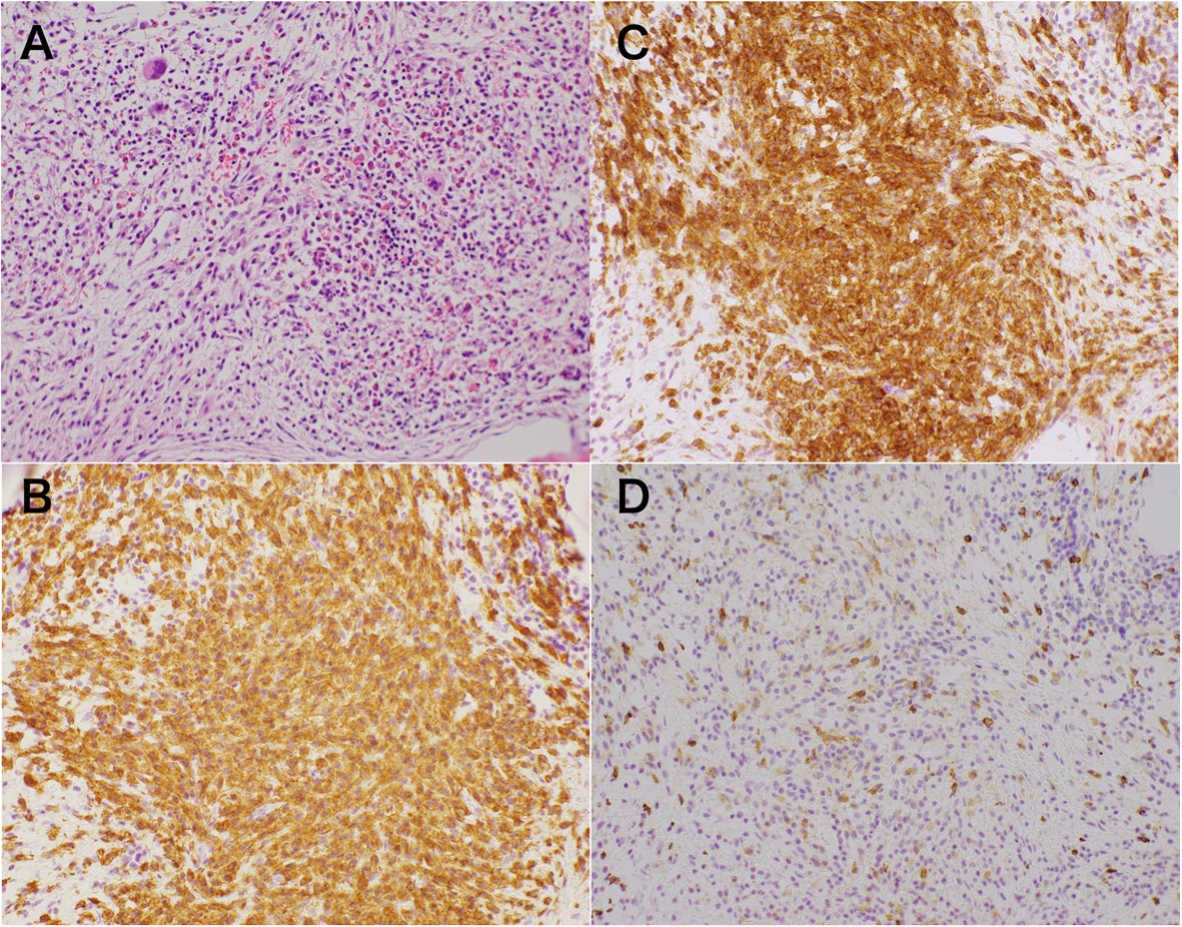
Bone marrow biopsy specimen showing marked infiltration by mast cells. **A** May-Giemsa stain, **B** CD117 stain, **C** CD25 stain, and **D** CD2 stain (original magnification, × 100)

**Fig. 3:**
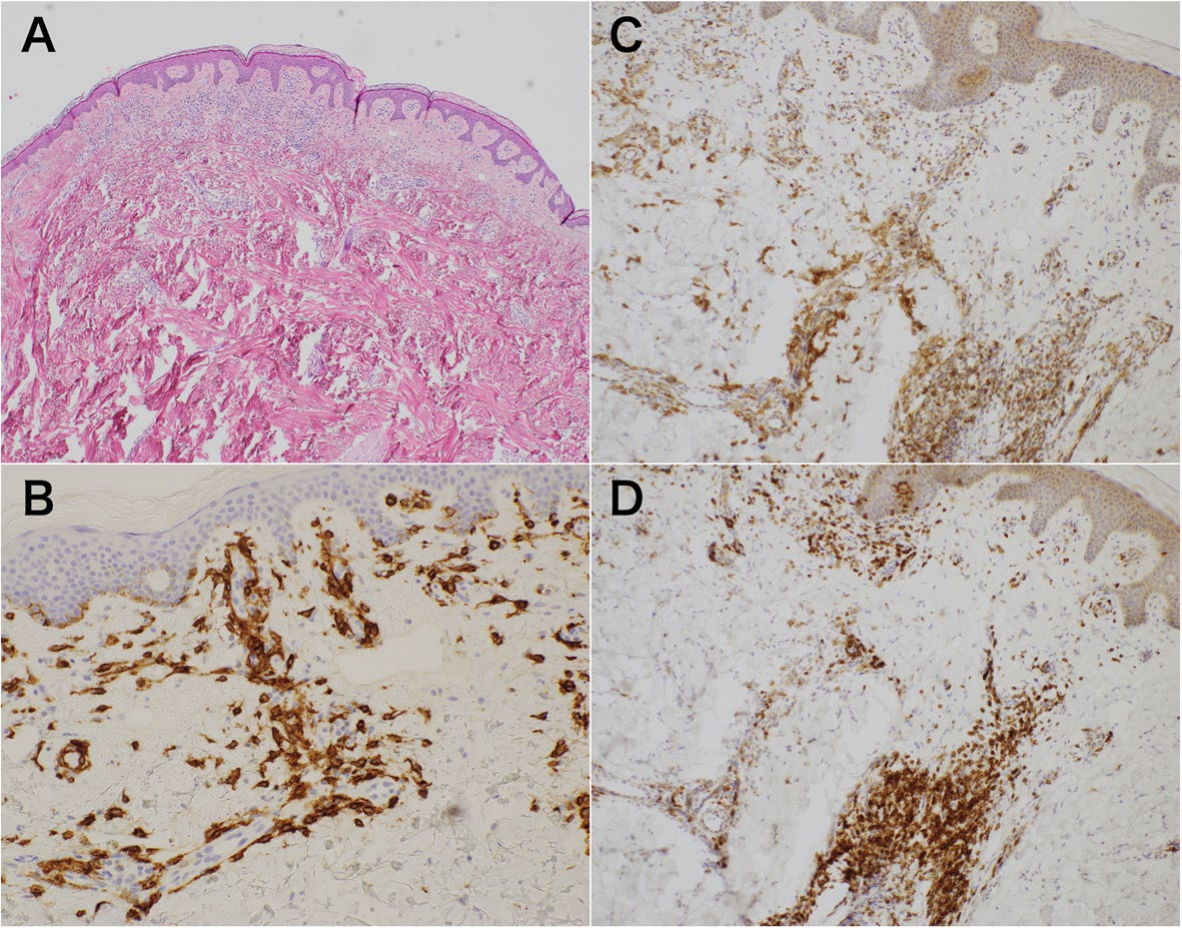
Biopsy specimen of a cutaneous rash on the abdomen, showing infiltration of the dermis by mast cells. **A** Hematoxylin and eosin stain, **B** CD117 stain, **C** CD25 stain, and **D** CD2 stain (original magnification, × 100)

He subsequently visited to the rheumatology and hematology department, where he underwent a bone marrow aspiration/biopsy; the aspiration resulted in dry tap like in previous attempt. The marrow obtained by biopsy contained abundant mast cells at least more than 80% (Fig. 2A) and was strongly positive for CD117 and CD25 and slightly positive for CD2 (Fig. 2B–D). These bone marrow findings suggested chronic mast cell leukemia (MCL), an advanced subtype of SM [5]; however, the diagnostic MCL criteria of WHO are based on marrow aspiration, not by biopsied specimen. A biopsy sample was also obtained from an area of cutaneous MPCM rash on his abdomen. Histopathologic examination of this rash specimen showed marked infiltration of mast cells positive for CD117, CD25, and CD2 (Fig. 3). A KIT point mutation at codon 816 mutation (D816V) was not confirmed in his peripheral blood. After SM diagnosis being confirmed, the patient was taken care in the hands of hemato-oncologists.

## Discussion and conclusions

Idiopathic anaphylaxis is a diagnosis of exclusion when no apparent cause of anaphylaxis can be identified. Previous studies report that 6.5–59% of episodes of anaphylaxis are idiopathic, although most of these episodes diagnosed as idiopathic are due to incomplete medical interviews or patient unawareness of allergens. Important differential diagnoses of the patients with anaphylactic episodes include any types of allergies, pigeon tick bite, wheat-dependent exercise-induced anaphylaxis, systemic capillary leak syndrome, and mast cell disorders [2–4]. Other differential diagnoses include “allergy mimics” such as asthma masquerading as anaphylaxis, panic attacks, globus hystericus, vocal cord dysfunction, vasoactive amine intolerance, carcinoid syndrome, and pheochromocytoma [2–4].

Diagnosis of SM is challenging because most symptoms and signs involving several organs are generally nonspecific [6, 7]. According to the 2016 diagnostic criteria of the World Health Organization (WHO) [5], diagnosis of SM requires one major criterion and one minor criterion or three minor criteria. The major criteria include a multifocal dense infiltrate of mast cells (≥ 15 on aggregate) in bone marrow biopsies and/or in sections of other extracutaneous organ(s). The minor criteria are as follows: (a) > 25% of all mast cells must be atypical (type 1 or type 2) on bone marrow smear or be spindle shaped in infiltrates detected on sections of visceral organs; (b) the presence of a KIT point mutation at codon 816 in the bone marrow or another extracutaneous organ; (c) mast cells in bone marrow, blood, or another extracutaneous organ must be positive for CD2 and/or CD25; and (d) baseline serum tryptase levels must be > 20 ng/ml, although this criterion is not considered valid in patients with an unrelated myeloid neoplasm. Because the present patient fulfilled the major and three of the minor diagnostic criteria, he was diagnosed with SM.

MCL, an advanced variant of SM, requires the presence of ≥ 20% atypical and immature mast cells in the bone marrow, or 10% in the blood, on good-quality smears [12]. MCL could not be definitively diagnosed in the present patient because of no blasts in the peripheral blood and the lack of bone marrow smears due to dry tap marrow.

Patients with SM also show skeletal involvement, such as osteopenia/osteoporosis with bone pain [13], as observed in the present patient. Bone disease is known to be the common imaging feature in patients with SM, and its detection may help diagnose the disease. In addition, it was reported skeletal involvement may become a prognostic factor [13]. However, it should be most emphasized that gastrointestinal symptoms have been reported in more than 50% of SM patients [8, 9]. Direct infiltration of the gastrointestinal tract by clonal mast cells with a KIT point mutation has not been well documented [8], although biopsies of the intestines and mast cell staining of biopsied tissues are required for a diagnosis of SM in patients with acute abdomen [7, 8]. Moreover, SM-associated gastrointestinal symptoms are due to the secondary effects of chemical mediators secreted by mast cells. Gastrointestinal involvement as part of SM in the present patient was diagnosed not by intestinal biopsy but by a constellation of evidence consisting of bone marrow/skin biopsy findings as well as abdominal CT findings showing edematous and swollen intestines, with underlying long medical history of repeated anaphylactic episodes. The reason for no previous SM diagnosis in the present case is unclear, but it may have been due to the lack of recognition of mast cell activation disorders as one of the causes of acute abdomen by the patient’s prior physicians.

Treatment of SM at the time of acute anaphylactic episodes should include supportive care, avoidance of triggers, and administration of oxygen, antihistamines, leukotriene antagonists, H2 antagonists, and/or proton pump inhibitors and steroids. The present patient was appropriately and successfully treated at the emergency room, and after SM diagnosis being made, the patient was transferred for chemotherapy by hemato-oncologists.

In summary, this report describes acute abdomen in a patient with SM in an emergency setting. Despite repeated anaphylactic episodes over 30 years, SM in this patient was undiagnosed prior to visiting our hospital, because previous evaluations somehow failed to confirm the mast cell activation disease. The patient’s symptoms were resolved at the emergency room by administration of oxygen, histamine (H1 and H2) receptor antagonists, and corticosteroid. Since SM in this patient was evaluated as an advanced variant (probable MCL), the patient needed chemotherapy. Generally, the risk of severe mast cell-activation anaphylactoid episodes should be reduced by adequate treatment. If not appropriately treated, the disease might be lethal. In addition, in cases of an advanced variant of SM, targeted drugs such as tyrosine kinase and/or KIT inhibitors besides chemotherapy could be effective [14]. Physicians should keep in mind anaphylactoid gastrointestinal diseases due to mast cell activation when seeing patients with acute abdomen at the emergency room.

## Data Availability

All data generated or analyzed during this study are included in this published article.

## Abbreviations

SM: Systemic mastocytosis
MCL: Mast cell leukemia
CT: Computed tomography
MPCM: Maculopapular cutaneous mastocytosis
